# Infant with Clinical Evidence of Pulmonary Hypoplasia: A Case Report

**DOI:** 10.7759/cureus.1298

**Published:** 2017-05-30

**Authors:** Talia Glasberg, Paige Jackson, Zdena Pavlova, Srikumar Nair

**Affiliations:** 1 Neonatology, Children's Hospital of Los Angeles; 2 Pathology, Children's Hospital of Los Angeles

**Keywords:** pulmonary hypoplasia, respiratory failure, diaphragmatic eventration

## Abstract

Pulmonary hypoplasia is the incomplete development of lung tissue. A reduced number of lung cells, airways, and alveoli is the hallmark and can be seen unilaterally or in both lungs. The diagnosis, however, is usually made upon pathologic examination. Here we have presented a case of a term infant presenting with severe hypoxemic respiratory failure. Despite optimizing medical and respiratory management, the infant passed away at 22 hours of life. On autopsy, she was discovered to have bilateral diaphragmatic eventrations, which is a rare cause of secondary pulmonary hypoplasia. She also was found to have some other minor abnormalities on autopsy but no unifying cause for the eventrations and other abnormalities was elucidated.

## Introduction

Pulmonary hypoplasia can be caused by multiple different mechanisms and is the end result of deficient lung development leading to a decreased number of small airways, alveoli, and pulmonary blood vessels [1]. Pulmonary hypoplasia can be grouped into primary pulmonary hypoplasia, in which it is the intrinsic lung development that is abnormal, or secondary pulmonary hypoplasia, in which the lung development is compromised secondary to another abnormality. Oligohydramnios is a common cause of secondary pulmonary hypoplasia and can be due to renal malformations, early and prolonged amniotic fluid leak, placental abnormalities, or intrauterine growth restriction. Lesions that occur in the thorax can cause compression of the lungs and lead to hypoplasia, as is seen with congenital diaphragmatic hernia, cystic lung disease, or extreme cardiomegaly. Absence or abnormal diaphragmatic activity, which can prevent chest wall expansion and breathing movements, can also lead to pulmonary hypoplasia, as in our case [1]. Bilateral diaphragmatic eventration is not common and physicians must keep the diagnosis in mind for children with severe respiratory failure with presumed pulmonary hypoplasia that cannot be attributed to oligohydramnios or mass occupying lesions.

## Case presentation

Baby L was a 37 1/7 week female infant born to a 26yo G3P3 mother via cesarean section for failure to progress and non-reassuring fetal heart tones. Pregnancy was complicated by new-onset polyhydramnios at 35 weeks. At 36 3/7 weeks, the amniotic fluid index was noted to be 40.6 cm, increasing to 41.2 cm prior to delivery. Previous ultrasounds were reportedly normal. The mother was followed with serial biophysical profiles with scores of 8/8 at her last two visits prior to delivery.

Maternal prenatal labs were unremarkable and past medical history was significant only for kidney stones, status post lithotripsy.

Spontaneous rupture of membranes occurred nine hours prior to delivery, the mother remained afebrile, and no antibiotics were administered. At delivery, the infant was noted to have low tone and no spontaneous respiratory effort, which did not improve with positive pressure ventilation. She was consequently intubated in the delivery room with Apgar of 5/7/7 at 1, 5 and 10 minutes, respectively. No cord gases were obtained. Birth weight was 2795 g (50th%), head circumference 35.5 cm (97th%), and length 51 cm (90th%). She was admitted to the neonatal ICU (NICU) at the birth hospital secondary to respiratory failure requiring mechanical ventilation. Initially, she required moderate respiratory support with conventional ventilation at a rate of 30, peak inspiratory pressure (PIP) 20, positive end-expiratory pressure (PEEP) 5 with 100% FiO2. Initial oxygen saturation was 51%. Umbilical arterial and venous catheters were inserted, and initial arterial blood gas (ABG) showed significant respiratory and metabolic acidosis with pH 6.63 and PaO2 of 46.7. PCO2, bicarbonate and base deficit were unreadable. By report, chest x-ray showed poorly expanded lungs. She was then placed on the high frequency oscillatory ventilator (HFOV) with amplitude of 22, hertz of 12, mean airway pressure (MAP) of 12 and 100% FiO2 with improvement of oxygen saturations to 80%. Subsequent ABGs continued to show significant respiratory and metabolic acidosis with pH 6.72, pCO2 212.9, PaO2 71, bicarbonate 26.9 and base deficit of -14.7. The MAP was increased to 15, with minimal improvement. She received two normal saline boluses for poor perfusion and was started on dopamine for hypotension. Ampicillin and gentamicin were started for presumed sepsis. Initial chemistries, complete blood count, and coagulation studies were reportedly unremarkable, apart from acidosis. Our institution was then contacted for extracorporeal membrane oxygenation (ECMO) evaluation. Inhaled nitric oxide (iNO) was started at 20 ppm by our transport team with no improvement in oxygen saturations. During transport, dopamine was escalated from 5 to 15 mcg/kg/hr to maintain systemic blood pressure in the normal range to encourage pulmonary blood flow and improve oxygen saturations.

The infant arrived at 8 hours of age and at that time the physical exam was notable for an axillary temperature of 36.9, BP of 52/36 with a mean arterial pressure of 41 and she was tachycardic with a heart rate of 170 bpm. She was orally intubated on HFOV and on a radiant warmer. The anterior fontanel was open, soft and flat, her ears were slightly cupped and low set with a triangular shaped face and a mildly webbed neck. Pupils were reactive to light and she opened her eyes spontaneously. There was no gag present on suctioning. She had symmetric chest wiggle on the oscillator. Capillary refill was less than 3 seconds. The abdomen was scaphoid appearing with no hepatosplenomegaly. Umbilical lines were in place. She had a patent, normally placed anus and normal appearing female genitalia. No rashes or bruises noted on the skin but she was pale. Bilateral fingers were contracted and there was diffuse hypotonia with decreased bulk of her muscles.

**Figure 1:**
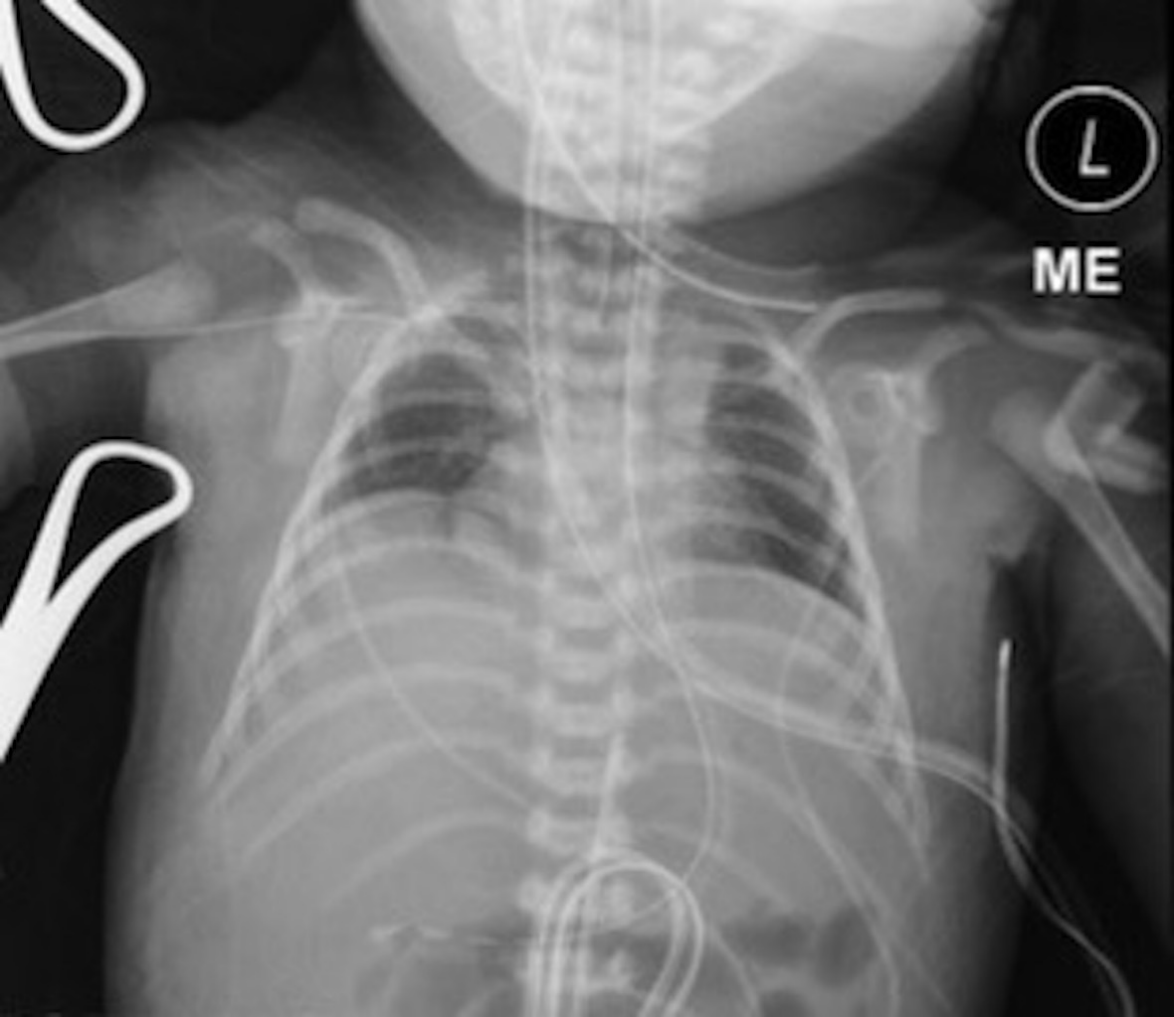
AP chest x-ray

High-frequency ventilation was resumed upon admission with moderate settings (amplitude of 36, hertz of 8, MAP 15, 100% FiO2). iNO was continued at 20 ppm with initial ABG showing improvement (pH = 7.12, pCO2 72, PaO2 40, bicarbonate 24, base deficit -7.1). Preductal saturations on admission were 82% and post ductal saturations were 85%. A chest x-ray confirmed low lung volumes (Figure 1). Fentanyl and versed continuous infusions were initiated for sedation and comfort. A cranial ultrasound was obtained that showed no gross abnormalities or evidence of intraventricular hemorrhage. Repeat laboratory studies were unremarkable.

An echocardiogram was obtained that showed normal cardiac anatomy, but a moderately dilated and hypertrophied right ventricle with severely depressed function. Left ventricular size was normal, though the function was moderately reduced. There was bi-directional shunting (mostly right to left) across a widely stretched patent foramen ovale, tricuspid valve insufficiency with a peak gradient of 38 mmHg, and a moderate to large patent ductus arteriosis with mostly right to left shunting. Right ventricular pressures were estimated to be extremely elevated. Milrinone and sildenafil continuous infusions were started for the treatment of acute pulmonary hypertension.

On arrival, mean arterial pressures were >35 but within a few hours she began to suffer from refractory hypotension. Dopamine was escalated to 30 mcg/kg/min, dobutamine was started and titrated up to 20 mcg/kg/min, and epinephrine was started and titrated up to 0.2 mcg/kg/min. Hydrocortisone was given to treat possible adrenal insufficiency.

Given suspicion for pulmonary hypoplasia and concern for renal abnormalities, an abdominal ultrasound was obtained that showed the presence of bilateral, normal appearing kidneys, hepatomegaly, a malrotated and displaced spleen and fluid-filled bowel loops. Repeat chemistries were unremarkable except for continued acidosis. She had brisk urine output throughout the hospitalization.

Despite maximal therapy with multiple pressors, fluid resuscitation, and optimizing ventilator settings, she continued to have persistent hypoxemia, hypotension, and acidosis. Given the persistent hypoexpansion on x-ray, there was a concern for primary pulmonary hypoplasia. With this information in mind, the medical team concluded that the patient was not an ECMO candidate. The parents decided to redirect care towards palliation and comfort, aggressive care was withdrawn, and the patient was extubated and held by her father. At the time of death, the infant was 22 hours old.

**Figure 2:**
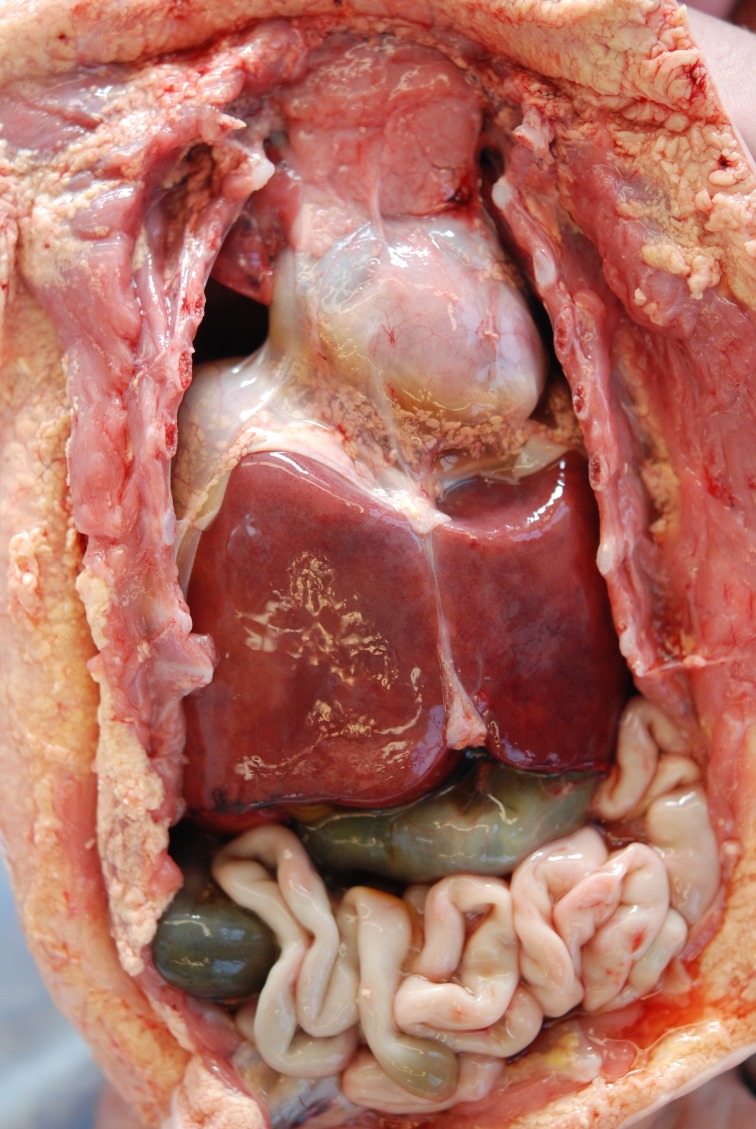
Frontal view of chest and abdomen showing elevated position of small lungs and the translucent, fibrous appearance of diaphragm

Gross autopsy examinations revealed the right lung to have an incomplete horizontal fissure with a hypoplastic upper lung. The left lung had no fissure. Both lungs were very small. The diaphragm was intact and at the level of the fourth and fifth ribs (Figure 2). The liver was transverse but normally lobated. The spleen was bilobed and slightly smaller than expected. The uterus and vagina exhibited a didelphys configuration with a double vagina.

**Figure 3:**
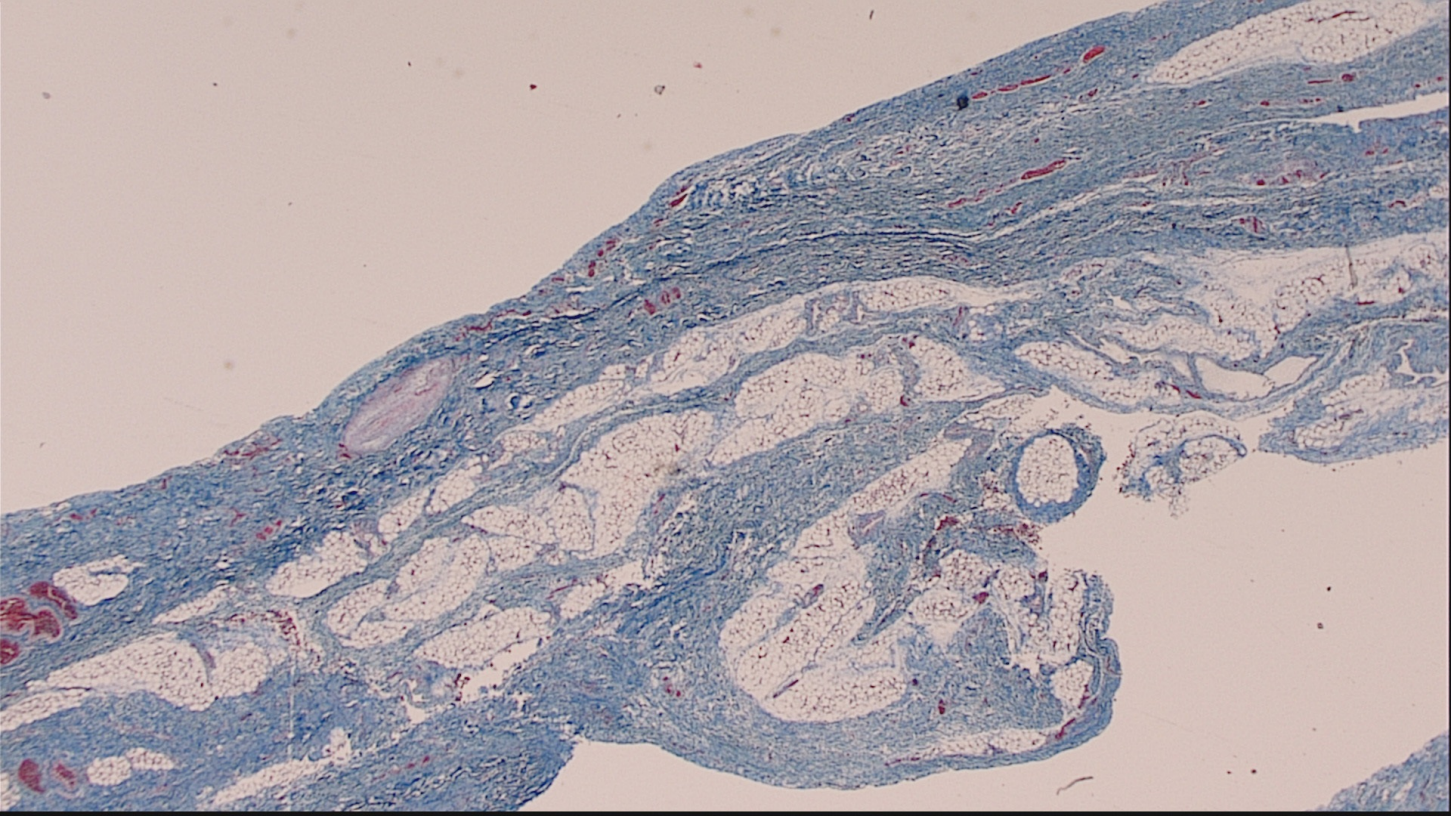
Trichrome stain showing absence of muscle fibers in diaphragm

On microscopic exam, the lungs were found to have markedly dysmorphic lung parenchyma with complex enlarged but collapsed bronchi with deficient musculature. On trichrome staining where muscle fibers should appear red and fibrous tissue blue, there was found to be a virtual absence of muscle fibers in the bronchi. The lungs showed decreased alveoli to bronchial tissue and large bronchi were seen near the vicinity of the pleura, which can be seen in hypoplastic lungs. The pulmonary arteries had markedly thickened walls and the lungs appeared immature with thickened septa. Sections of the sternocleidomastoid muscle, psoas muscle and quadriceps showed slightly small muscle fibers but no structural abnormalities. Using specific staining techniques, no increased fibrosis or fat accumulation was found. Glycogen distribution in the muscle sections was normal. Nicotinamide dinucleotide diaphorase (NADH), succinate dehydrogenase (SDH), and cytochrome oxidase (COX) staining were non-specific and adenosine triphosphatase (ATPase) stains did not reveal a specific diagnosis. The diaphragm was found to have a virtual absence of muscle fibers with trichrome staining as well. The normal muscle was replaced with dense fibrous tissue, adipose tissue, and blood vessels (Figure 3).

Blood was sent for survival motor neuron 1 (SMN 1) deletion analysis to evaluate for possible spinal muscular atrophy, which returned negative. A microarray, lactate and pyruvate levels, and plasma amino acids were also normal.

## Discussion

We have presented a case of a 37 week female infant born to a mother with a pregnancy complicated only by polyhydramnios in the last two weeks prior to delivery, who presented with intractable hypoxic respiratory failure, minimal lung expansion and elevated diaphragms on x-ray. She was found to have marked pulmonary hypoplasia, significantly decreased musculature of the bronchial tree as well as virtually no musculature of the diaphragm on autopsy. Primary pulmonary hypoplasia was the initial working diagnosis for this patient, as she had no history or outward exam findings to suggest other causes for her respiratory failure and minimal lung expansion. However, upon autopsy, the absence of muscle fibers in the diaphragm leading to diaphragmatic eventration, gives a potential cause of her pulmonary hypoplasia.

Congenital diaphragmatic eventration, a condition where the diaphragm is either completely or partly replaced with fibroelastic tissue, is usually localized or unilateral and has rarely been documented as bilateral [2]. The most common cause of unilateral diaphragmatic eventration is phrenic nerve injury from delivery. Many cases are discovered on chest x-ray when the diaphragm is seen in an elevated position. Eventrations can be asymptomatic if the affected area is small and can cause increasing respiratory compromise as the defects become larger or are bilateral. Our patient had bilateral eventration and associated pulmonary hypoplasia that caused fatal respiratory failure. The association between diaphragmatic disorders and pulmonary hypoplasia is thought to be due to the lack of fetal breathing that occurs when the diaphragms are not functioning correctly; this could also be a potential cause of polyhydramnios as seen in this case. Usually when diaphragm dysfunction is this severe, it is found to be due to nervous system damage (i.e., phrenic nerve injury) or a congenital neuropathy or myopathy [1].

Goldstein and Reid published a case of bilateral phrenic nerve agenesis associated with diaphragmatic amyoplasia and pulmonary hypoplasia [3]. Their case reported on a term infant with significant respiratory failure unresponsive to maximal measures with small lungs seen on chest x-ray. On autopsy, phrenic nerves could not be identified and the diaphragm was intact but had virtually no muscular components and was predominantly fibrous tissue. The infant had normally lobed lungs but both were small and poorly aerated. On microscopic exam, they found evidence of pulmonary artery hyperplasia. They also discuss a prior case of membranous diaphragms and lung hypoplasia that was thought to be related to congenital rubella, but there was no suspicion for this in our patient.

Elberg, et al. describe a similar situation with congenital bilateral eventration in a pair of twins [4]. The infants were born at 36 weeks, both developed severe respiratory failure and expired. Of note, similar to our case, the pregnancy was noted to be complicated by polyhydramnios. On autopsy, both infants were found to have a total absence of muscle in the diaphragms and the lungs were hypoplastic and atelectatic. The etiology of the diaphragmatic eventration was unknown.

Renault, et al. also reported on two cases of diaphragmatic eventration without obvious cause [5]. Their first patient was a child who was born at term and had hypotonia and respiratory failure. A biopsy of the diaphragm showed almost no muscle fibers and the diaphragm was noted to have minimal movement on ultrasound. This child underwent a neuromuscular workup (electromyogram, nerve conduction studies) and was not found to have any other neuromuscular deficits outside the diaphragm. The child survived the newborn period but died at 11 months from asthma. The second case described is a term infant with severe respiratory failure who was born after a pregnancy complicated by polyhydramnios. The diaphragms were surgically plicated after a biopsy revealed virtually no muscle fibers. This child had a neuromuscular workup similar to the previous child and no abnormalities were detected.

After autopsy and laboratory analysis of our patient were completed, we did not find an underlying neuromuscular diagnosis for this infant, although she did not undergo electromyogram analysis or genetic testing other than the microarray and spinal muscular atrophy testing. The infant was hypotonic and could have warranted further myopathy or neuropathy workup had she survived longer. Her muscles, on biopsy, did appear to have fibers that were a little smaller than expected, but they did not have any obvious abnormalities and no stains were able to elucidate a diagnosis.

## Conclusions

Bilateral diaphragmatic eventration is a rare cause of secondary pulmonary hypoplasia. This diagnosis must be considered in patients with severe respiratory failure and suspected pulmonary hypoplasia when the history is inconsistent with more common causes, such as oligohydramnios or space occupying lesions.

## References

[1] Crowley M (2015). Neonatal respiratory disorders. Fanaroff and Martin’s Neonatal-Perinatal Medicine. Volume 2. 10th edition.

[2] Parker T, Kinsella J (2012). Respiratory failure in the term newborn. Avery’s Diseases of the Newborn. 9th edition.

[3] Goldstein J, Reid L (1980). Pulmonary hypoplasia resulting from phrenic nerve agenesis and diaphragmatic amyoplasia. J Pediatr.

[4] Elberg J, Brok K, Pedersen S (1989). Bilateral eventration of the diaphragm in a pair of male twins. J Pediatr Surg.

[5] Renault F, Nicot F, Liptai Z (2008). Congenital diaphragm weakness without neuromuscular disease. Muscle Nerve.

